# Supplementary material for: A virus-encoded protein suppresses methylation of the viral genome through its interaction with AGO4 in the Cajal body
Source: eLife. 2020 Oct 16;9:e55542. doi: 10.7554/eLife.55542 (PMC7567605; doi:10.7554/eLife.55542)
Supplement: Supplementary file 1. [file elife-55542-supp1.docx]

|  |  | **Supplementary File 1. Values of IR methylation in independent experiments and replicates.** | | | | | | | | | | | | |  |  |
| --- | --- | --- | --- | --- | --- | --- | --- | --- | --- | --- | --- | --- | --- | --- | --- | --- |
|  |  | **Percentage of methylated cytosines in the intergenic region (IR) of TYLCV in local infection assays with TYLCV or the V2 null mutant (Figure 4A).** | | | | | | | | | | | | |  |  |
|  |  | **%** | **3 dpi / TYLCV** | | | **3 dpi / TYLCV-V2null** | | | **9 dpi / TYLCV** | | | **9 dpi / TYLCV-V2null** | | |  |  |
|  |  |  | 1st | 2nd | 3rd | 1st | 2nd | 3rd | 1st | 2nd | 3rd | 1st | 2nd | 3rd |  |  |
|  |  | **CG** | 0.92 | 0 | 1.11 | 61.8 | 62.12 | 47.22 | 0 | 0.46 | 5.32 | 72 | 77.58 | 78.57 |  |  |
|  |  | **CHG** | 0 | 0 | 4 | 62.5 | 70 | 43.33 | 0 | 0 | 6.77 | 76.15 | 84.82 | 80 |  |  |
|  |  | **CHH** | 0 | 0.19 | 1.11 | 63.02 | 57.97 | 49.07 | 0.28 | 0.81 | 7.3 | 73.55 | 72.7 | 73.74 |  |  |
|  |  | **All** | 0.166 | 0.143 | 1.402 | 62.75 | 59.93 | 48.17 | 0.208 | 0.666 | 6.902 | 73.54 | 74.79 | 75.24 |  |  |
|  |  |  |  | **Percentage of methylated cytosines in the intergenic region (IR) of TYLCV in local infection assays with the V2 null mutant in *AGO4*-silenced or control plants (Figure 4B).** | | | | | | | | |  |  |  |  |
|  |  |  |  | **%** | **TYLCV-V2null / TRV-EV** | | | | **TYLCV-V2null / TRV-*NbAGO4*** | | | |  |  |  |  |
|  |  |  |  |  | 1st | 2nd | 3rd | 4th | 1st | 2nd | 3rd | 4th |  |  |  |  |
|  |  |  |  | **CG** | 59.52 | 55.73 | 58.78 | 45.61 | 46.75 | 11.56 | 34.72 | 34.05 |  |  |  |  |
|  |  |  |  | **CHG** | 64.28 | 50.64 | 58.79 | 47.69 | 48.33 | 13.33 | 36.42 | 34.34 |  |  |  |  |
|  |  |  |  | **CHH** | 61.11 | 52.8 | 55.55 | 45.26 | 48.14 | 11.21 | 33.33 | 32.02 |  |  |  |  |
|  |  |  |  | **All** | 61.142 | 53.113 | 56.451 | 45.573 | 47.916 | 11.489 | 33.892 | 32.622 |  |  |  |  |
| **Percentage of methylated cytosines in the intergenic region (IR) of TYLCV in systemic infection assays with TYLCV or the V2 null mutant in *AGO4*-silenced or control plants (Figure 4C).** | | | | | | | | | | | | | | | | |
| **%** | **TYLCV / TRV-EV** | | | | **TYLCV / TRV-*NbAGO4*** | | | | **TYLCV-V2null / TRV-EV** | | | | **TYLCV-V2null / TRV-*NbAGO4*** | | | |
|  | 1st | 2nd | 3rd | 4th | 1st | 2nd | 3rd | 4th | 1st | 2nd | 3rd | 4th | 1st | 2nd | 3rd | 4th |
| **CG** | 49.55 | 6.34 | 7.71 | 27.84 | 34.6 | 0 | 0 | 0 | 56.29 | 71.79 | 60.58 | 69.04 | 39.76 | 43.82 | 29.53 | 56.96 |
| **CHG** | 49.39 | 6.42 | 7.77 | 31.87 | 35.61 | 0.86 | 0 | 0 | 52.66 | 66.92 | 57.41 | 73.8 | 38.94 | 45.21 | 26.31 | 56.36 |
| **CHH** | 44.34 | 6.55 | 8.26 | 30.43 | 31.85 | 0.18 | 0.07 | 0.08 | 53.05 | 67.61 | 55.75 | 65.05 | 39.47 | 42.68 | 25.01 | 54.16 |
| **All** | 45.79 | 6.504 | 8.115 | 30.11 | 32.72 | 0.217 | 0.055 | 0.058 | 53.6 | 68.3 | 56.79 | 66.65 | 39.47 | 43.14 | 25.96 | 54.89 |
|  |  | **Percentage of methylated cytosines in the intergenic region (IR) of TYLCV in systemic infection assays with TYLCV or the V2 null mutant in *Nbcoilin*-silenced or control plants (Figure 7E).** | | | | | | | | | | | | |  |  |
|  |  | **%** | **TYLCV / TRV-EV** | | | **TYLCV / TRV-*Nbcoilin*** | | | **TYLCV-V2null / TRV-EV** | | | **TYLCV-V2null / TRV-*Nbcoilin*** | | |  |  |
|  |  |  | 1st | 2nd | 3rd | 1st | 2nd | 3rd | 1st | 2nd | 3rd | 1st | 2nd | 3rd |  |  |
|  |  | **CG** | 2.77 | 0 | 0 | 3.59 | 0.3 | 0.27 | 18.14 | 46.6 | 71.79 | 5.98 | 7.07 | 50.39 |  |  |
|  |  | **CHG** | 1.66 | 0 | 0.52 | 4.11 | 0.55 | 0 | 22.66 | 53.88 | 73.07 | 6.92 | 7.27 | 55 |  |  |
|  |  | **CHH** | 1.54 | 0.13 | 0.21 | 3.6 | 0.07 | 0.06 | 18.88 | 45.09 | 65.52 | 4.91 | 7.71 | 50.74 |  |  |
|  |  | **All** | 1.778 | 0.1 | 0.21 | 3.651 | 0.166 | 0.1 | 19.133 | 46.247 | 67.411 | 5.311 | 7.552 | 51.111 |  |  |
