# Supplementary material for: A virus-encoded protein suppresses methylation of the viral genome through its interaction with AGO4 in the Cajal body
Source: eLife. 2020 Oct 16;9:e55542. doi: 10.7554/eLife.55542 (PMC7567605; doi:10.7554/eLife.55542)
Supplement: Supplementary file 2. [file elife-55542-supp2.docx]

**Supplementary File 2. Exclusive unique peptide count of NRPE1 co-immunoprecipitated with NbAGO4-1 in the presence or absence of V2 as identified by AP-MS.** Results from two independent biological replicates are shown.

| **Identified protein name** | **Molecular Weight** | **Exclusive unique peptide count in each IP sample** | | | |
| --- | --- | --- | --- | --- | --- |
|  |  | **GFP + TYLCV** | **NbAGO4-1 +TYLCV** | **NbAGO4-1 + TYLCV-V2null** | **NbAGO4-1 + TYLCV-V2null + V2** |
| **3xFLAG-NbAGO4-1** | 105 kDa | 0 / 0 | 27 / 40 | 36 / 44 | 37 / 52 |
| **FLAG-GFP** | 30 kDa | 7 / 4 | 0 / 0 | 0 / 0 | 0 / 0 |
| **V2** | 13 kDa | 0 / 0 | 1 / 1 | 0 / 0 | 1 / 6 |
| **DNA directed RNA polymerase E subunit 1 (NRPE1)** | 224 kDa | 0 / 2 | 2 / 23 | 8 / 18 | 7 / 21 |
