# Supplementary material for: A virus-encoded protein suppresses methylation of the viral genome through its interaction with AGO4 in the Cajal body
Source: eLife. 2020 Oct 16;9:e55542. doi: 10.7554/eLife.55542 (PMC7567605; doi:10.7554/eLife.55542)
Supplement: Supplementary file 3. [file elife-55542-supp3.docx]

| **Supplementary File 3. List of primers used for cloning in this study.** | | |
| --- | --- | --- |
| Plasmid name | Vector name (Source) | Primer sequence |
| (1) TOPO cloning | | |
| TOPO-*NbAGO4-1* (with stop codon) | pENTR/D-TOPO (Invitrogen) | F: CACCATGGCTGAAGAAGACAATGG R: TTAACAGAAGAACATGGAACTAGAAACAT |
| TOPO-*NbAGO4-2* (with stop codon) | pENTR/D-TOPO (Invitrogen) | F: CACCATGGCTGAAGAAGATAAGAAT R: TCAACAAAAGAACATGGAACTGGAAACT |
| TOPO-*SlAGO4a* (with stop codon) | pENTR/D-TOPO (Invitrogen) | F: CACCATGGCTGAAGAAGAAACAAATG R: TTAGCAAAAGAACATAGAACTGGAAAC |
| TOPO-*SlAGO4b* (with stop codon) | pENTR/D-TOPO (Invitrogen) | F: CACCATGGCTGAAGAAGAGAATGGT R: TCAACAGAAGAACATGGAGCTAGCAAC |
| TOPO-*SlAGO4d* (with stop codon) | pENTR/D-TOPO (Invitrogen) | F: CACCATGGCCTCTTCAAAAGATGAAG R: TTAGCAGAAGAACATTGAACTGCGAACAT |
| TOPO-*SlWRKY75* (with stop codon) | pENTR/D-TOPO (Invitrogen) | F: CACCATGGAGAATTATGCAACAATATTTCC R: AAAGGAATTATAGATTTGCATTTGAC |
| TOPO-VIGS-*NbAGO4* | pENTR/D-TOPO (Invitrogen) | F: CACCATGATTCTTGGAATGGA R: GTGACCACTTCTCATCAAGA |
| TOPO-VIGS-*Nbcoilin* | pENTR/D-TOPO (Invitrogen) | F：CACCTTTATAATGAAGACGGATCTCTGGA  R：GAAGGCCATTGTAGGGCCCAG |
| (2) BP reaction | | |
| pDONR221-P1P4-V2 / pDONR221-P1P4-V2_L76S_ | pDONR221-P1P4 (Invitrogen) | F: GGGGACAAGTTTGTACAAAAAAGCAGGCTTAATGTGGGACCCACTTCTAAATGAA R: GGGGACAACTTTGTATAGAAAAGTTGGGTTCAGGGCTTCGATACATTCTGT |
| pDONR221-P1P4-*NbAGO4-1* | pDONR221-P1P4 (Invitrogen) | F: GGGGACAAGTTTGTACAAAAAAGCAGGCTTAATGGCTGAAGAAGACAATGGTGG R: GGGGACAACTTTGTATAGAAAAGTTGGGTTTAACAGAAGAACATGGAACTAGAAACATT |
| pDONR221-P1P4-*NbAGO4-2* | pDONR221-P1P4 (Invitrogen) | F: GGGGACAAGTTTGTACAAAAAAGCAGGCTTAATGGCTGAAGAAGATAAGAAT R: GGGGACAACTTTGTATAGAAAAGTTGGGTTCAACAAAAGAACATGGAACTGGAAACT |
| pDONR221-P1P4-*SlAGO4a* | pDONR221-P1P4 (Invitrogen) | F: GGGGACAAGTTTGTACAAAAAAGCAGGCTTAATGGCTGAAGAAGAAACAAATG R: GGGGACAACTTTGTATAGAAAAGTTGGGTTTAGCAAAAGAACATAGAACTGGAAAC |
| pDONR221-P1P4-*SlAGO4b* | pDONR221-P1P4 (Invitrogen) | F: GGGGACAAGTTTGTACAAAAAAGCAGGCTTAATGGCTGAAGAAGAGAATGGTG R: GGGGACAACTTTGTATAGAAAAGTTGGGTTCAACAGAAGAACATGGAGCTAGCAACG |
| pDONR221-P3P2-V2 / pDONR221-P3P2-V2_L76S_ | pDONR221-P3P2 (Invitrogen) | F: GGGGACAACTTTGTATAATAAAGTTGGAATGTGGGACCCACTTCTAAATGAA R: GGGGACCACTTTGTACAAGAAAGCTGGGTGGGGCTTCGATACATTCTGTATATTCT |
| (3) Infusion cloning | | |
| pCambia1300-3xFLAG-*NbAGO4-1* | pCAMBIA1300 (Cambia) | 1300-FLAG-F: CGGGGGACTGTCGACTCTAGAATGGACTACAAAG 1300-NbAGO4-1-FLAG-R: TGTCTTCTTCAGCCATCTTATCGTCATCGTCCTTG |
|  |  | NbAGO4-1-F: ATGGCTGAAGAAGACAATGGTGG 1300-NBAGO4-1-R: TCGATCAATCAGGTACCCGGGTTAACAGAAGAACATGGAACT |
| (4) V2 null mutagenesis | | |
| TOPO-TYLCV-V2null | TOPO-TYLCV (Rosas-Diaz et al., 2018) | F: GTCTTGCAATATGTAGGACCCACTTCTAAATGAATTTCCTG R: CAGGAAATTCATTTAGAAGTGGGTCCTACATATTGCAAGAC |
| TOPO-TYLCV-V2_L76S_ | TOPO-TYLCV (Rosas-Diaz et al., 2018) | F: GCCGAAGGCTGAATCTCGACAGC R: GCTGTCGAGATTCAGCCTTCGGC |
| TOPO-V2_L76S_ | TOPO-V2 (Wang et al., 2017a) | F: GCCGAAGGCTGAATCTCGACAGC R: GCTGTCGAGATTCAGCCTTCGGC |
