# Supplementary material for: A virus-encoded protein suppresses methylation of the viral genome through its interaction with AGO4 in the Cajal body
Source: eLife. 2020 Oct 16;9:e55542. doi: 10.7554/eLife.55542 (PMC7567605; doi:10.7554/eLife.55542)
Supplement: Supplementary file 4. [file elife-55542-supp4.docx]

| **Supplementary File 4. List of plasmids used in this study.** | | |
| --- | --- | --- |
| Plasmid name | Entry vector name (Source) | Binary vector (Source) |
| (1) Confocal microscopy | | |
| RFP-*NbAGO4-1* | TOPO-*NbAGO4-1* (This study) | pGWB555 (Nakagawa et al., 2007) |
| RFP-*NbAGO4-2* | TOPO-*NbAGO4-2* (This study) | pGWB555 (Nakagawa et al., 2007) |
| RFP-*SlAGO4a* | TOPO-*SlAGO4a* (This study) | pGWB555 (Nakagawa et al., 2007) |
| RFP-*SlAGO4b* | TOPO-*SlAGO4b* (This study) | pGWB555 (Nakagawa et al., 2007) |
| RFP-*SlAGO4d* | TOPO-*SlAGO4d* (This study) | pGWB555 (Nakagawa et al., 2007) |
| CFP-Fibrillarin | pDONR207-*Fibrillarin* (Kim et al., 2007b) | pGWB545 (Nakagawa et al., 2007) |
| (2) Luciferase Complementation Imaging assay | | |
| V2-N-luc | TOPO-V2 (Wang et al., 2017a) | pGWB-N-luc (Yu et al., 2019) |
| C-luc-*NbAGO4-1* | TOPO-*NbAGO4-1* (This study) | pGWB-C-luc (Yu et al., 2019) |
| C-luc-*NbAGO4-2* | TOPO-*NbAGO4-2* (This study) | pGWB-C-luc (Yu et al., 2019) |
| C-luc-*SlAGO4a* | TOPO-*SlAGO4a* (This study) | pGWB-C-luc (Yu et al., 2019) |
| C-luc-*SlAGO4b* | TOPO-*SlAGO4b* (This study) | pGWB-C-luc (Yu et al., 2019) |
| C-luc-*SlWRKY75* | TOPO-*SlWRKY75* (This study) | pGWB-C-luc (Yu et al., 2019) |
| (3) Immunoprecipitation | | |
| 3xHA-*NbAGO4-1* | TOPO-*NbAGO4-1* (This study) | pGWB515 (Nakagawa et al., 2007) |
| 3xHA-*NbAGO4-2* | TOPO-*NbAGO4-2* (This study) | pGWB515 (Nakagawa et al., 2007) |
| 3xHA-*SlAGO4a* | TOPO-*SlAGO4a* (This study) | pGWB515 (Nakagawa et al., 2007) |
| 3xHA-*SlAGO4b* | TOPO-*SlAGO4b* (This study) | pGWB515 (Nakagawa et al., 2007) |
| 3xHA-*SlAGO4d* | TOPO-*SlAGO4d* (This study) | pGWB515 (Nakagawa et al., 2007) |
| *V2_L76S_*-GFP | TOPO-V2_L76S_ (This study) | pGWB505 (Nakagawa et al., 2007) |
| (4) Virus-induced gene silencing | | |
| TRV-*NbAGO4* | TOPO-VIGS-*NbAGO4* (This study) | pTRV2-Gw (Taylor et al., 2012) |
| TRV-*Nbcoilin* | TOPO-VIGS-*Nbcoilin* (This study) | pTRV2-Gw (Taylor et al., 2012) |
| (5) BiFC | | |
| V2-nYFP + cYFP-empty | pDONR221-P3P2-V2 (This study) | pBiFC-2in1-CN  (Grefen & Blatt, 2012) |
| V2-nYFP + cYFP-V2 | pDONR221-P3P2-V2 (This study) +  pDONR221-P1P4-V2 (This study) | pBiFC-2in1-CN  (Grefen & Blatt, 2012) |
| V2-nYFP + cYFP-*NbAGO4-1* | pDONR221-P3P2-V2 (This study) + pDONR221-P1P4-*NbAGO4-1* (This study) | pBiFC-2in1-CN  (Grefen & Blatt, 2012) |
| V2-nYFP + cYFP-*NbAGO4-2* | pDONR221-P3P2-V2 (This study) + pDONR221-P1P4-*NbAGO4-2* (This study) | pBiFC-2in1-CN  (Grefen & Blatt, 2012) |
| V2-nYFP + cYFP-Sl*AGO4a* | pDONR221-P3P2-V2 (This study) + pDONR221-P1P4-Sl*AGO4a* (This study) | pBiFC-2in1-CN  (Grefen & Blatt, 2012) |
| V2-nYFP + cYFP-Sl*AGO4b* | pDONR221-P3P2-V2 (This study) + pDONR221-P1P4- Sl*AGO4b* (This study) | pBiFC-2in1-CN  (Grefen & Blatt, 2012) |
| V2_L76S_-nYFP + cYFP-empty | pDONR221-P3P2-V2_L76S_ (This study) | pBiFC-2in1-CN  (Grefen & Blatt, 2012) |
| V2_L76S_-nYFP + cYFP-V2_L76S_ | pDONR221-P3P2-V2_L76S_ (This study) + pDONR221-P1P4-V2_L76S_ (This study) | pBiFC-2in1-CN  (Grefen & Blatt, 2012) |
| V2_L76S_-nYFP + cYFP-*NbAGO4-1* | pDONR221-P3P2-V2_L76S_ (This study) + pDONR221-P1P4-*NbAGO4-1* (This study) | pBiFC-2in1-CN  (Grefen & Blatt, 2012) |
| V2_L76S_-nYFP + cYFP-*NbAGO4-2* | pDONR221-P3P2-V2_L76S_ (This study) + pDONR221-P1P4-*NbAGO4-2* (This study) | pBiFC-2in1-CN  (Grefen & Blatt, 2012) |
