# Supplementary material for: A virus-encoded protein suppresses methylation of the viral genome through its interaction with AGO4 in the Cajal body
Source: eLife. 2020 Oct 16;9:e55542. doi: 10.7554/eLife.55542 (PMC7567605; doi:10.7554/eLife.55542)
Supplement: Supplementary file 5. [file elife-55542-supp5.docx]

| **Supplementary File 5. List of primers used for qPCR or qRT-PCR in this study.** | | | |
| --- | --- | --- | --- |
| Target gene | Forward Primer (5’-3’)  Reverse Primer (5’-3’) | Primer pair efficiency | Reference |
|  |  |  |  |
| (1) RT-qPCR | | | |
| *NbAGO4-1* | F: TGGTGAAGTGCAAACATCGG | 106.7% | This study |
|  | R: TTGCAATGAGACCAACGAGC |  |  |
| *NbAGO4-2* | F: GCCTTGCATCAATGTTGGGA | 101.1% | This study |
|  | R: TGAGGGCATTGCTTAGGACT |  |  |
| *NbAGO6* | F: GCTAGTGCACCGGAAAATGT | 88.6% | This study |
|  | R: ATAGAGGTCGCAGTCGTGCT |  |  |
| *NbTubulin* | F: CAAGATGCTACTGCAGACGAG | 99.7% | Liu et al., 2012 |
|  | R: CTGGAAGTTGTGGTTTTGGC |  |  |
| *Nbcoilin* | F: TGTGCCCAAGGAAGGTCTTGTG | 96.6% | This study |
|  | R: GGCATCAGCAGAACTCTATTTGCTTCA |  |  |
| *SlAGO4a* | F: TCAAATGGGACAGTGGATGA | 95.9% | This study |
|  | R: TTTCTCCTCGAGCTTTGGAA |  |  |
| *SlAGO4b* | F: GGGTCAATGGATGAAATTCG | 98.7% | This study |
|  | R: GGGGCAATTGAGGAACAGTA |  |  |
| *SlAGO4c* | F: TCAGGGATGGTGTCAGTGAA | 103.9% | This study |
|  | R: GGGCGACCACTTTTCATCTA |  |  |
| *SlAGO4d* | F: AAGGCAAAGAGGATGCTGAA | 95.8% | This study |
|  | R: CAACGTTAATGCAGGGGAGT |  |  |
| *SlActin* | F: CCTCAGCACATTCCAGCAG | 92.1% | Exposito-Rodriguez et al., 2008 |
|  | R: CCACCAAACTTCTCCATCCC |  |  |
| (2) qPCR for virus accumulation | | | |
| Rep | F: TGAGAACGTCGTGTCTTCCG | 94.80% | Wang et al., 2017b |
|  | R: TGACGTTGTACCACGCATCA |  |  |
| *ITS* | F: ATAACCGCATCAGGTCTCCA | 101.1% | Mason et al., 2008 |
|  | R: CCGAAGTTACGGATCCATTT |  |  |
| (3) qPCR following ChIP or RIP in *Nicotiana benthamiana* | | | |
| IR | F: CGGTGTCCCTCAAAGCTCTA | 88.1% | This study |
|  | R: TAGCCATTAGGTGTCCAGGT |  |  |
| V2 | F: AATCATTTCCACGCCCGTCT | 89.2% | This study |
|  | R: GTACGTCCATGATCGTCGCT |  |  |
| *Actin* | F: CGGAATCCACGAGACTACATAC | 108.1% | Maimbo et al., 2010 |
|  | R: GGGAAGCCAAGATAGAGC |  |  |
